# Integrative Analysis of Drug Co-Prescriptions in Peritoneal Dialysis Reveals Molecular Targets and Novel Strategies for Intervention

**DOI:** 10.3390/jcm14113733

**Published:** 2025-05-26

**Authors:** Michail Evgeniou, Paul Perco, Fabian Eibensteiner, Markus Unterwurzacher, Andreas Vychytil, Rebecca Herzog, Klaus Kratochwill

**Affiliations:** 1 Division of Pediatric Nephrology and Gastroenterology, Department of Pediatrics and Adolescent Medicine, Comprehensive Center for Pediatrics, Medical University of Vienna, Währinger Gürtel 18-20, 1090 Vienna, Austria; n1231826@students.meduniwien.ac.at (M.E.); fabian.eibensteiner@meduniwien.ac.at (F.E.); markus.unterwurzacher@medunwien.ac.at (M.U.); 2 Delta 4 GmbH, Alserstrasse 23, 1080 Vienna, Austria; paul.perco@delta4.ai; 3 Department of Internal Medicine IV, Medical University Innsbruck, Anichstrasse 35, 6020 Innsbruck, Austria; 4 Division of Nephrology and Dialysis, Department of Medicine III, Medical University of Vienna, Währinger Gürtel 18-20, 1090 Vienna, Austria; andreas.vychytil@meduniwien.ac.at

**Keywords:** peritoneal dialysis, drug combination, network analysis, biological networks, drug repurposing

## Abstract

**Background/Objectives:** Peritoneal dialysis (PD) is a renal replacement therapy for patients with kidney failure. Managing PD patients often involves addressing a complex interplay of comorbidities and complications, necessitating the use of multiple medications. This study aimed to systematically characterize commonly co-prescribed drugs in PD and to identify novel drug combinations that may target dysregulated molecular mechanisms associated with PD’s pathophysiology. **Methods:** We analyzed clinical records from 702 PD patients spanning 30 years, encompassing over 5500 prescription points. Using network-based modeling techniques, we assessed drug co-prescription patterns, clinical outcomes, and longitudinal treatment trends. To explore potential drug repurposing opportunities, we constructed a molecular network model of PD based on a consolidated transcriptomics dataset and integrated this with drug–target interaction information. **Results:** We found commonly prescribed drugs such as furosemide, sucroferric oxyhydroxide, calcitriol, darbepoetin alfa, and aluminum hydroxide to be integral components of PD patient management, prescribed in over 30% of PD patients. The molecular-network-based approach found combinations of drugs like theophylline, fluoxetine, celecoxib, and amitriptyline to possibly have synergistic effects and to target dysregulated molecules of PD-related pathomechanisms. Two further distinct categories of drugs emerged as particularly interesting in our study: selective serotonin reuptake inhibitors (SSRIs), which were found to modulate molecules implicated in peritoneal fibrosis, and vascular endothelial growth factor (VEGF) inhibitors, which exhibit anti-fibrotic properties that are potentially useful for PD. **Conclusions:** This comprehensive exploration of drug co-prescriptions in the context of PD-related pathomechanisms provides valuable insights for opening future therapeutic strategies and identifying new targets for drug repurposing.

## 1. Introduction

In silico medicine (computational medicine) has emerged as a powerful tool in modern medicine, transforming the ways in which diseases are diagnosed, monitored, and treated [1,2]. By using advanced modeling approaches, machine learning, and big data, in silico approaches in nephrology enable personalized treatment strategies, improve the early detection of disease and drug-induced toxicity, help predict disease progression in chronic kidney disease (CKD), and enhance the understanding of complex renal pathophysiology [3,4,5,6,7].

Peritoneal dialysis (PD) is a well-established renal replacement therapy for patients with kidney failure, offering an alternative to hemodialysis. By utilizing the peritoneal membrane, PD allows for the removal of waste products and excess fluid from the body. Its advantages include improved quality of life and cost-effectiveness [8]. However, the long-term success of PD is often compromised by complications such as chronic peritoneal inflammation, fibrosis, infections, and angiogenesis. Peritonitis, a common complication caused by various factors, further exacerbates peritoneal damage [9]. Beyond these complications, PD patients face a significantly higher risk of mortality due to comorbidities such as cardiovascular disease and metabolic disorders, including hypovitaminosis, hypokalemia, and dyslipidemia [10,11]. The need to manage these comorbidities often results in polypharmacy, where multiple drugs are prescribed to address various conditions. While polypharmacy is essential for managing complex disease states, it also increases the risk of adverse drug reactions and poses challenges for effective clinical management [12].

Despite advancements in PD research, major limitations remain. While in vitro, ex vivo, and in vivo studies provide valuable mechanistic insights and identify therapeutic targets, clinical translation is challenging. Large-scale randomized trials are difficult due to PD’s multifactorial nature, the limited patient population, and high costs [13].

Computational methodologies such as co-prescription analysis, drug combination studies, network-based approaches, and systems biology offer significant potential to enhance disease understanding, explore polypharmacology, and advance personalized medicine by aligning treatments with patients’ molecular and clinical profiles [14,15,16,17,18,19,20,21,22,23]. Analysis of drug combinations represents a promising strategy for various diseases by utilizing complementary mechanisms of action. This approach enables synergistic effects, enhances efficacy, and facilitates the simultaneous targeting of multiple disease pathways [24,25].

We hypothesize that patterns of drug co-prescription in PD patients reflect underlying pathophysiological processes, and that systematic analysis of these combinations—when linked to molecular network models—may uncover novel therapeutic strategies and repurposing opportunities for PD. Here, we therefore aimed to analyze drug combinations prescribed to patients undergoing chronic PD treatment using one of the largest single-center PD patient databases, containing data on three decades of patients receiving PD therapy. To complement this clinical dataset, we employed computational approaches to investigate the mechanisms of action of these drug combinations and assess their potential to mitigate PD-associated pathomechanisms by integrating clinical data in a PD-associated molecular pathomechanism network analysis.

## 2. Materials and Methods

### 2.1. PD Patient Database

PD-BASE, a biobank managed by the Medical University of Vienna, encompasses comprehensive information on the drug prescriptions of 702 PD patients over the course of the past three decades. The drug entries are presented in relation to a baseline, denoting the period before the initiation of PD and the subsequent period thereafter. The dataset comprises 5835 distinct prescription data points over the course of PD treatment in these patients. Drugs that were prescribed during a particular appointment were consistently recorded in subsequent appointments until their discontinuation. In order to unify the dataset, only drug prescriptions during the first PD phase of each patient were analyzed (depending on the endpoint of a PD phase, patients may have subsequent PD phases, e.g., a new PD phase following failure of a kidney transplant). Patients were stratified by decade (1990–2022), based on their full treatment period recorded in the database, defined as the range from their first to their last drug prescription. For individuals whose treatment spanned multiple decades, we assigned them to the decade in which the majority of their prescription events occurred, reflecting the predominant period of clinical management. The study and retrospective analysis were approved by the local ethics committee of the Medical University of Vienna (EK 2035/2015) and conducted in accordance with the Declaration of Helsinki.

### 2.2. Drug Data Harmonization and Co-Prescription Analysis in PD Patients

Drugs were recorded in the database with their German brand or generic names, as originally documented during clinical routine. Each database entry represented a prescription point for a patient, for one or multiple different drugs. To avoid transfer errors in the database, we used a semi-automated 2-stage pipeline. We first filtered for correct entries by identifying individual drugs based on matches with their ATC codes. In the second step, for entries with typographical errors, we calculated the Jaro–Winkler score [26] for the string similarity between the drug names from the ATC system and the prescribed drugs, and we used it as a distance measure for subsequent hierarchical clustering (Ward’s method, tree cut at 0.2) of all drugs contained in the database [27]. Eventually, these clusters were manually checked to correct inaccuracies resulting from the original manual data entry process.

### 2.3. Co-Prescription Rate and Similarity of Co-Prescribed Drugs

Co-prescription rates were analyzed by recording the prescription points at which each patient was prescribed drug A and/or drug B. Next, the Jaccard coefficient (also known as the Tanimoto coefficient) [28] was calculated on the sets of individual patients and times where drug A and/or drug B were prescribed. This was carried out for all possible drug pairs, allowing for the quantification of the frequency with which a drug pair (A and B) was co-prescribed at the population level. The Jaccard coefficient provides a value ranging from 0 to 1, indicating the degree of similarity between the sets of drug A and drug B, with 1 indicating perfect similarity and 0 indicating no similarity. In order to describe the molecular similarity between drug A and B in all potential drug co-prescription pairs in this clinical PD cohort, we calculated the Jaccard coefficient for the drugs’ mechanism of action, their direct targets, their targeted molecular pathways, and their chemical structure.

Four different Jaccard coefficients were calculated to evaluate the similarity between drugs: Mechanism similarity: Based on literature-derived gene associations for each drug, as stored in Delta4’s Hyper-C platform as described in previous publications [29]. Each set A and B contained the genes associated with drug A’s and drug B’s mechanisms of action, respectively. Direct drug target similarity: Drugs with direct drug target information derived from the ChEMBL database [30], with similarity measured between drugs based on shared known direct target genes. Each set A and B contained the known direct target genes of drug A and drug B, respectively. Molecular pathway similarity: Using the Reactome database [31], a pathway enrichment analysis was performed on drug target genes. Pathways were considered to be relevant if they contained at least three target genes per drug and had an adjusted *p*-value < 0.05. Drug similarity was determined by the number of shared molecular pathways; therefore, each set A and B contained the pathways associated with drug A’s and drug B’s mechanisms of action, respectively. Structure similarity: Calculated using ECFP4 molecular fingerprints [32]. Two-dimensional structures in the SMILES format were downloaded from PubChem [33]. Each set A and B contained the ECFP4 molecular fingerprint for drug A and B, respectively. Finally, similarity was assessed based on ATC code hierarchy at the fourth ATC code level, reflecting a drug’s chemical/therapeutic/pharmacological subgroup. Each set A and B contained the ATC code up to the fourth level of each drug A and B, respectively.

### 2.4. Peritoneal Dialysis Molecular Model (“PD Disease Network”)

In a recently conducted meta-analysis of human transcriptomics data in the context of PD, we identified key dysregulated molecular processes and mechanisms [34]. The core set of differentially expressed genes (DEGs) of this dataset was used to build a network-based molecular model following established bioinformatics workflows [29,35]. In brief, DEGs were mapped onto a protein–protein dependency network of experimentally determined protein–protein interaction data from IntAct, BioGRID, and Reactome, complemented by computationally inferred protein–protein dependencies based on a set of predefined data sources [36]. From the resulting network, we extracted the connected subgraph of proteins sharing at least one edge with another protein from the list of PD DEGs. A workflow diagram of the integration of the clinical database, the “PD disease network”, and the co-prescription analysis are shown in Supplementary Figure S1.

### 2.5. Drug–Target Interactions from the Public Domain and Network Clustering

Drug–target interactions were obtained from ChEMBL (version 32, released August 2023), encompassing a total of 4068 drug entries [30]. For the network proximity analysis, we used only the drug targets associated with each drug. However, the database includes drug entries that share identical drug target information, which would lead to repetition of the same analysis. To reduce this redundancy, we applied a clustering algorithm based on shared drug–target interactions. An adjacency matrix using the drug target similarity score described above was constructed. In this matrix, we set all values not equal to one to zero, ensuring that drugs with identical drug targets would be grouped together. As a result, we obtained multiple disconnected networks of one or more drugs belonging to the previously established clusters. We continued our analysis using these clusters and maintained the information regarding which drugs belonged to which cluster in a separate dictionary.

### 2.6. Identification and Prioritization of Novel Drug Combinations Interfering with PD-Associated Molecular Processes

To explore potential novel drug compounds associated with PD complications, the distances between the drug target clusters (as described above) and the proteins associated with the PD molecular network were computationally determined. The SAveRUNer package in R (v4.2.1) was utilized to assess the network distances and significance of the drug clusters in relation to the PD molecular model [37], using a one-tailed z-test (random network distributions were generated using 1000 iterations), followed by *p*-value-based filtering (*p* < 0.05, no multiplicity correction). The resulting clusters were mapped back to drug molecules and compared with drug targets and proteins involved in the identified biological processes, as described in our previous study [34]. To qualify for further consideration, a drug combination had to meet the following criteria: a significant (*p* < 0.05) proximity score for the potential target, both drugs targeting distinct biological processes within the GO terms previously identified within the “PD disease network” (angiogenesis, sprout angiogenesis, positive regulation of angiogenesis, inflammatory response), an ATC similarity score of 0, and a low structural similarity score. We further focused on drug pairs in which each drug targets a different dysregulated molecule, with both molecules involved in the same biological process. Additionally, drugs already prescribed within the PD cohort were annotated, with a focus on combinations where either both drugs or at least one had been used in our patient cohort. A comprehensive literature review was then conducted to determine whether these drug combinations had been previously studied in the context of PD. The objective was to find documented evidence on their mechanisms of action, particularly in relation to PD-associated complications. This refined approach enabled a systematic analysis and prioritization of potential drug candidates by integrating statistical analysis with real-world clinical data.

### 2.7. Statistics

All statistical and bioinformatics analyses were conducted in R [38]. To compare time and drug usage in PD across different PD endpoints, we applied a two-tailed Kruskal–Wallis test, followed by a two-tailed Wilcoxon test as a post hoc analysis for pairwise group comparisons. Correlations between prescription points and numbers of prescribed drugs were analyzed using Spearman’s rank correlation. A *p*-value of <0.05 was considered statistically significant.

## 3. Results

**Figure 1:**
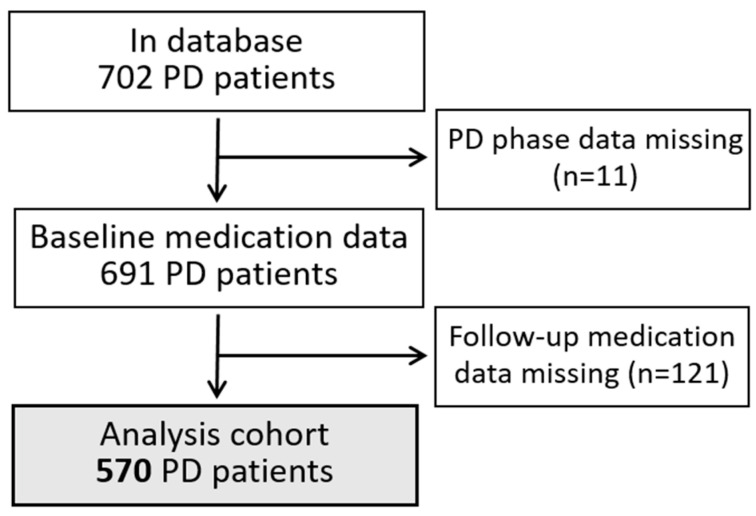
Patient flowchart of inclusion for analysis.

Of the 702 patients treated with PD and with medication records in the database, 570 had baseline and follow-up medication records and were therefore included in the analysis cohort (Figure 1).

These patients started PD therapy between March 1990 and July 2021. The demographic and therapy-related characteristics of the included patients are presented in Table 1. The dataset consisted of 5835 prescription points, with 547 unique drugs being prescribed. Each drug could be associated with multiple Anatomical Therapeutic Chemical (ATC) codes, resulting in 764 distinct ATC codes, corresponding to 323 different 4th level ATC codes.

During a mean duration of PD therapy (until an endpoint) of 25 [range 0.4–117.3] months, patients had a mean of nine [1,2,3,4,5,6,7,8,9,10,11,12,13,14,15,16,17,18,19,20,21,22,23,24,25,26,27,28,29,30,31,32,33,34,35,36,37,38,39,40,41,42] prescription points, where a mean of 12 [2,3,4,5,6,7,8,9,10,11,12,13,14,15,16,17,18,19,20,21,22,23] drugs were prescribed on each individual occasion, and they received a mean of 19 [2,3,4,5,6,7,8,9,10,11,12,13,14,15,16,17,18,19,20,21,22,23,24,25,26,27,28,29,30,31,32,33,34,35,36,37,38,39,40,41,42,43,44,45,46,47,48,49,50,51,52,53,54,55,56] different drugs over the complete course of the therapy. The mean time between prescription points was 2.8 [0–6.7] months.

**Figure 2:**
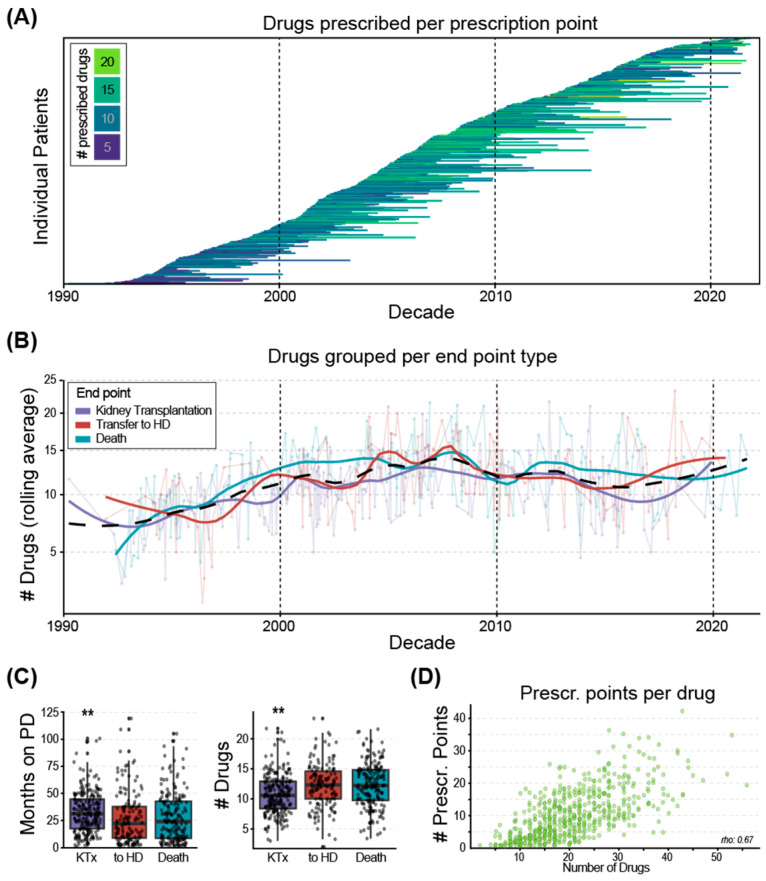
Drugs prescribed to individual PD patients over three decades: (**A**) Length of the lines: time range for which individual patients were treated with PD; color: mean unique drugs prescribed during the treatment period. (**B**) Over the decades of the investigated timeframe, the overall number of prescribed drugs per patient increased (black dashed line), and also when analyzed per endpoint type: solid violet: kidney transplantation; solid red: transfer to hemodialysis (HD); solid turquoise: death of the patient. (**C**) Black dots: individual patients; left: patients with the endpoint kidney transplantation (KTx) were treated with PD for longer compared to the other two endpoints, while they were prescribed a lower number of drugs (** *p* < 0.001, Wilcoxon signed-rank test, adjusted). (**D**) Number of prescribed drugs is correlated with the prescription points per patient; each dot represents an individual patient.

The dataset encompassed 32 years of drug prescriptions, which were grouped into three decades (1990–1999, 2000–2009, and 2010–2022). An upward trend was observed in the number of prescribed drugs per prescription point during the first decade, but independent of the later endpoint, namely, kidney transplantation, transfer to hemodialysis, or death (Figure 2A,B). Although the effect sizes were small (below 0.2), the results showed that the duration of PD treatment and the number of different drugs prescribed differed between patients later receiving kidney transplantation, transferred to HD, or who died on PD (Kruskal–Wallis *p*-value = 0.000002, *p*-value = 0.0008, respectively). Post hoc comparisons demonstrated that patients who eventually received a kidney transplantation were treated with PD for longer compared to those who died (*p* = 0.005) and those who were transferred to HD (*p* = 0.002). Similarly, those who received a kidney transplantation were prescribed fewer different drugs compared to those who died (*p* = 0.00003) and those who were transferred to HD (*p* = 0.00004) (Figure 2C). The duration of PD therapy was positively correlated with the number of prescribed drugs (rho = 0.67, *p* < 0.0001) (Figure 2D).

### 3.1. Co-Prescribed Drugs During PD Therapy Show Three Distinct Patterns

**Figure 3:**
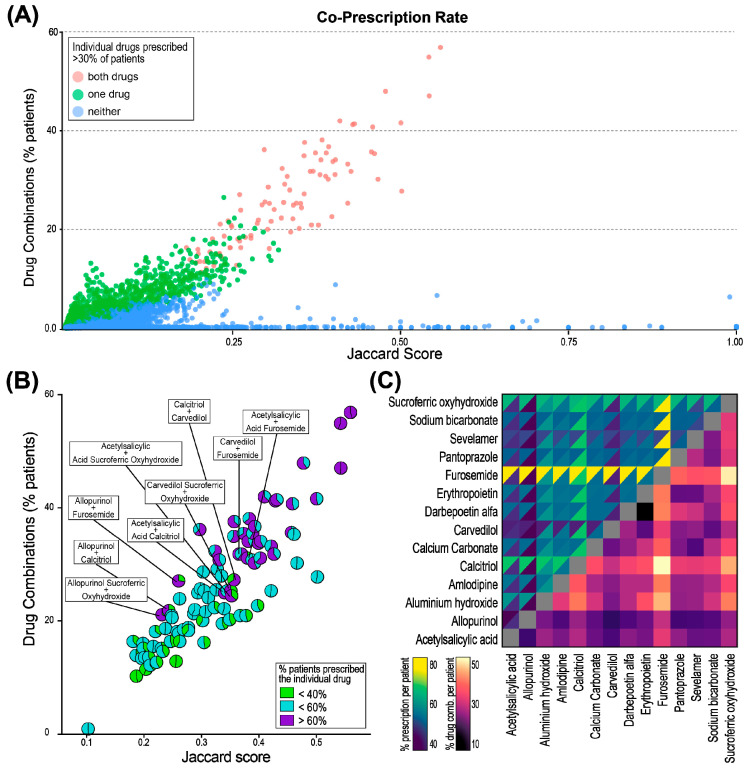
Three groups of different drug co-prescription patterns, based on the frequency of co-prescription and individual prescription to patients: (**A**) Color indicates prescription to >30% of patients: red: both drugs; green: one of the two drugs; blue: neither of the drugs. (**B**) Further detail on the group with both drugs > 30% (red dots in (**A**)). The fractions of the circles depict the ratio of the percentages of the two drugs. (**C**) Heatmap of the most prescribed drug combinations. Left side: individual drug prescriptions; triangles: percentage of prescriptions for each individual drug. Right side: percentage of patients co-prescribed a specific drug combination.

To identify the most commonly prescribed drug combinations in PD treatment, we analyzed the number of patients receiving each combination and the co-prescription rate between drug pairs, comparing their frequencies. A total of 21,553 unique drug combinations were identified, which were clustered into three distinct groups (Figure 3A). The largest group (*n* = 16,105) consisted of combinations where neither of the two drugs was prescribed to more than 30% of the patients. These drug combinations also showed higher Jaccard scores (up to 1, indicating that they were co-prescribed in 100% of patients and times). Many of these combinations are typical drug combinations specific to certain diseases (e.g., human immunodeficiency virus (HIV) infection, Parkinson’s disease, hepatitis C infection, or combinations of vitamins), highlighting already-established drug combinations.

In the second-largest group (*n* = 5357), only one of the two drugs was prescribed to more than 30% of the patients. This reflects drug pairs with low-to-moderate rates of prescription or rare occurrences of co-prescription, likely tailored to the specific conditions of individual patients rather than being broadly applicable to the majority of PD patients.

The third group (*n* = 91) comprised drug combinations in which both drugs were highly prescribed individually (to more than 30% of all patients). This group included drugs such as furosemide, sucroferric oxyhydroxide, calcitriol, darbepoetin alfa, and aluminum hydroxide (Figure 3B). Notably, some combinations, like furosemide and calcitriol, were prescribed in more than 30% of PD patients, with a co-prescription rate of 0.51. This means that both drugs were individually prescribed to over 30% of patients, and in 50% of those patients they were prescribed together (Figure 3C).

Similarity metrics were calculated for all drugs co-prescribed in the same patient at least once (Table S1). A correlation analysis was performed to identify relationships between drug combinations, with a specific focus on drug pairs sharing similarity at the 4th ATC level (chemical level). A significant but weak positive correlation (r = 0.24, *p* < 0.0001) between structural similarity and ATC similarity was found. Pathway and gene similarity scores showed a stronger correlation (r = 0.70, *p* < 0.0001). Despite the high overall correlation, some drug combinations exhibited low gene similarity while maintaining high pathway similarity. This occurs when drugs act on shared molecular pathways but target different genes. Prominent examples are metoclopramide–tropisetron (to manage nausea), levofloxacin–rabeprazole (for example for treatment of *H. pylori* infections), and estradiol–progesterone (for managing symptoms of menopause).

### 3.2. Network Analysis for Drugs Targeting Biological Processes in the “PD Disease Network”

A network analysis was conducted to calculate the proximity between subnetworks formed by the direct drug targets from drugs collected from the ChEMBL database and a previously established molecular process-based “PD disease network” [34]. Human protein–protein interaction data [36] served as the background network.

Among the analyzed drugs, 666 exhibited a proximity score ranging from 0 to 0.88 and *p* < 0.05 (Table S2). Of these, 41 drugs were already prescribed within our PD cohort (Table 2).

### 3.3. Drug Shortlisting in PD: Targeting Angiogenesis and Inflammation

**Figure 4:**
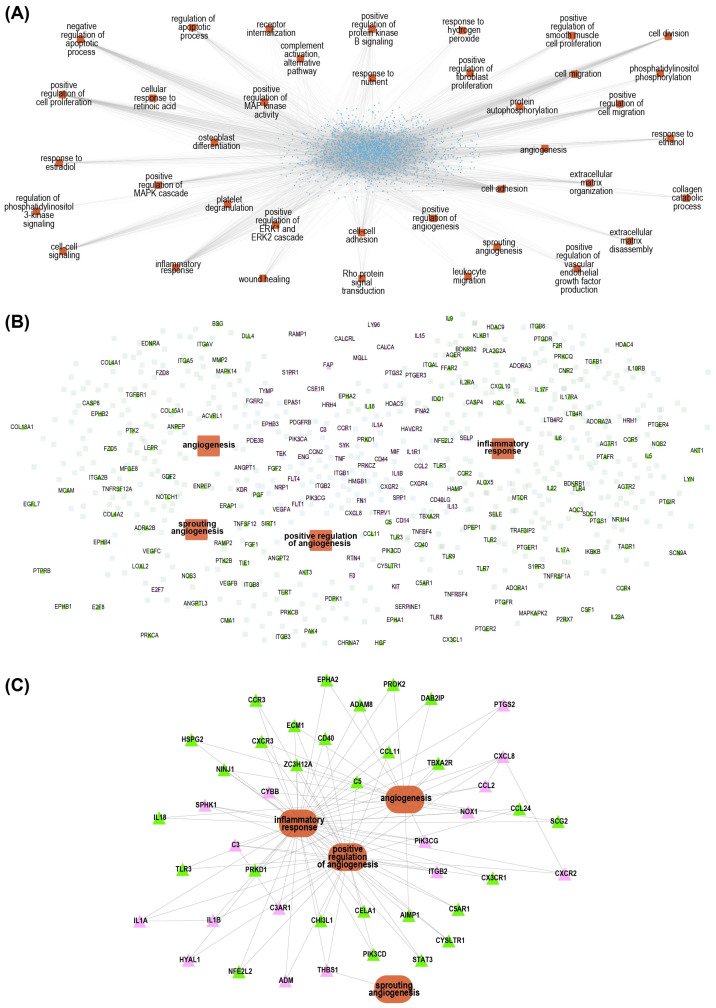
Construction of the PD model: (**A**) In a previous study, we developed a comprehensive PD model using a curated set of dysregulated molecules associated with the most pertinent complications (blue). From these molecules, we elucidated the key dysregulated biological processes (orange), with a particular focus on those relevant to inflammation and angiogenesis. (**B**) To enhance the model’s precision, we incorporated genes associated with the clinically most important pathophysiological processes, inflammation and angiogenesis, sourced from the Gene Ontology (GO) database (green). Drug targets based on information from the ChEMBL database are shown as pink triangles. (**C**) Additionally, we pinpointed specific drug targets that simultaneously influence both inflammation and angiogenesis biological processes. GO-derived information is in green, ChEMBL-derived information is in pink, and biological processes are in brown.

Next, we focused on drugs with demonstrated relevance in key biological processes already described in PD’s pathophysiology (i.e., the GO terms angiogenesis and inflammation) [10,34,39,40] (Figure 4A).

This investigation identified 43 drugs that interact with proteins known to be dysregulated within the PD disease subnetwork of angiogenesis and inflammation (Figure 4B). Five of the identified drugs (pazopanib, theophylline, rofecoxib, meloxicam, and celecoxib) are already used in our PD patient cohort; the remaining 38 drugs have not yet been prescribed (Table S3, Figure 4C). From the proximity analysis, we found that certain drugs targeting SLC6A4 and SLC6A2 (which have been associated with selective serotonin reuptake inhibitors (SSRIs) [41,42,43,44]) were closely related to the “PD disease network”. Twenty-one individual drugs had a proximity score below 0.5 (Table S2), indicating a significant connection. Although these drugs did not directly link SSRIs to inflammation or fibrosis, their proximity to the PD network, along with the fact that nine of these drugs have already been prescribed to PD patients (paroxetine, escitalopram, citalopram, sertraline, fluoxetine, venlafaxine, amitriptyline, duloxetine, and milnacipran (Table 2)), led to further exploration of the potential connections.

We also found that VEGF inhibitors (such as abicipar pegol, bevacizumab, brolucizumab, pegaptanib sodium, ranibizumab, and aflibercept) were strongly represented in our results (Table S2), with proximity scores between 0 and 0.5 and a *p*-value of 0.03.

### 3.4. Synergistic Potential of Drug Combinations in PD: Structural and Pharmacological Similarity Analysis

**Figure 5:**
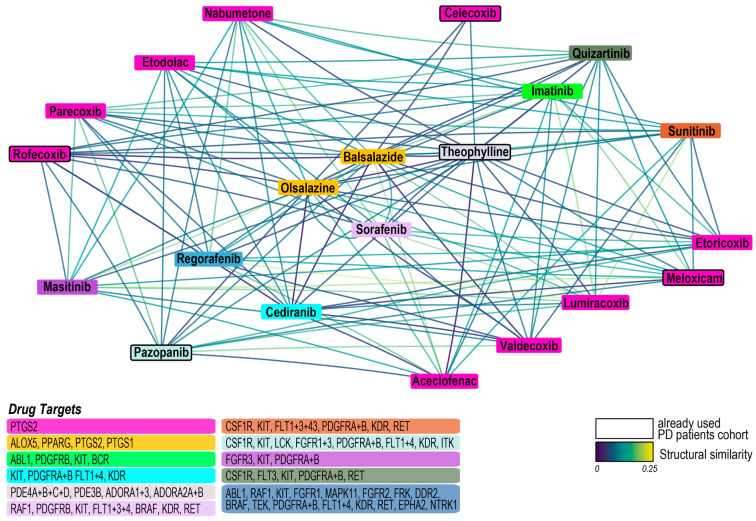
Drugs closely aligned with the PD disease network: All drugs concurrently targeting biological processes related to angiogenesis and inflammation through shared or direct targets. Each color corresponds to drugs sharing identical direct drug targets (table bottom left with targeted genes); color-coded lines indicate the structural similarities between paired drugs; frames: already prescribed in our PD patient cohort.

Structurally similar drugs are more likely to exhibit comparable or identical functions [45]. Building on recent findings [23], we hypothesized that drugs with significant proximity in targeting biological processes within the PD model may possess synergistic potential. We identified 21 drugs with the potential to form drug combinations with an ATC similarity score of 0 that exhibited significant proximity to the PD disease network (Figure 5). Of these, 5 have already been prescribed to PD patients, while 16 represent novel candidate drugs to target angiogenesis and inflammation in PD.

## 4. Discussion

Despite advancements in PD research, major treatment limitations remain. While in vitro, ex vivo, and in vivo studies provide valuable mechanistic insights and identify therapeutic targets, clinical translation is challenging. Clinical (cohort) studies are often small, lacking statistical power for definitive conclusions. Large-scale randomized trials are difficult due to PD’s multifactorial nature, limited patient population, and high costs [13].

Despite clinically and economically relevant advantages, PD is associated with complications such as chronic inflammation, peritonitis, and ultrafiltration failure. Rare but insufficiently understood events of encapsulating peritoneal sclerosis (EPS) impact not only patients’ outcomes but also the choice of PD as renal replacement therapy [46]. Kidney failure patients treated with PD most often present with comorbidities requiring the use of complex medication regimens, potentially leading to polypharmacy. PD patients represent a specific subgroup of CKD patients with common phenotypic patterns, including PD-specific complications and comorbidities, which may—due to their complex nature—be an attractive case for the analysis of drug combinations. Analyses of drug co-prescriptions have shown promise in tackling complex diseases characterized by intricate molecular interactions, including different types of cancer, infectious diseases, and cardiovascular diseases [47,48]. In particular, analyzing co-prescription patterns enhances the understanding of polypharmaceutical trends and aids in suggesting innovative drug combinations to study in clinical trials to facilitate repurposing [49,50]. Therefore, we first characterized co-prescribed drugs in a large longitudinal cohort of 570 PD patients. The mean number of drugs prescribed to PD patients increased over the 1990s but reached a plateau in the early 2000s, potentially indicating advancements and refinements in PD and CKD therapy. This observed increase in the number of prescribed drugs per patient may have different causes. On the one hand, documentation, and especially the availability of information of drugs intermittently prescribed by primary care physicians, may have improved during the 1990s, thus creating an artefact. On the other hand, the availability of drugs for and studies investigating drugs in kidney failure patients has increased over the last few decades, potentially also changing prescription patterns. However, with the data available from our records, this cannot be narrowed down to its specific cause. Multiple health conditions or the need to address various symptoms simultaneously can result in a plethora of co-prescribed drugs, which may elevate the risk of drug interactions, adverse effects, and non-adherence to the prescribed medication. Drug prescription in PD is generally under-discussed when compared with hemodialysis. However, a few studies have analyzed the burden of polypharmacy and tried to describe drug regimens in both dialysis treatments [51,52,53].

In our cohort, on average, patients with more prescription data points had more drugs prescribed per prescription point, and patients with a favorable endpoint (KTx) were prescribed fewer drugs than patients with less favorable endpoints (transfer to HD or death). Whether these observations mean that patients with a lower number of prescribed drugs and/or KTx during their course of PD therapy had fewer comorbidities or had an overall better health status, or that reduced polypharmacy favors good outcomes, remains to be elucidated in future studies.

Drug co-prescription offers a prospect of synergistic interactions between medications, potentially yielding additive therapeutic effects that surpass individual drug efficacy. Detecting such interactions may uncover new indications for existing drug combinations, broadening their therapeutic scope.

Moreover, drug combinations may aid in overcoming drug resistance, reducing side effects, and thereby improving adherence. By targeting multiple disease pathways simultaneously, drugs with complementary mechanisms of action offer novel therapeutic opportunities. Identifying such complementary mechanisms can be approached through molecular structure comparisons or direct target analysis, although the latter is often less informative. To systematically assess co-prescribed drugs, several similarity metrics were introduced in our analysis. Network analysis identified 666 drugs with proximity to PD-associated pathophysiological processes, of which 41 were prescribed in our cohort. Among drugs targeting the GO terms angiogenesis and inflammation, 43 were linked to dysregulated proteins, with 21 specifically affecting both angiogenesis and inflammatory responses. Of these, five were prescribed in our cohort. A key tool for drug classification and comparison is the ATC system, which categorizes medications based on their therapeutic properties and organ/system of action.

Drugs commonly prescribed in our cohort, such as furosemide, also have a potential for adverse reactions when combined with other medications. Here, we provide an entry point to further investigate the landscape of potential drug interactions based not only on drug co-prescription rates but extending to other pharmacological similarity measures. Pairing that information with existing information regarding drug–drug interactions may be useful to evaluate the safety of drugs prescribed in combination [54].

We recently developed a method to generate a molecular fingerprint for the mode of action of drugs by identifying dysregulated genes relevant to PD and mapping them onto a protein–protein interaction network constructed from both in vitro and in silico data from various sources. This model helps narrow the focus on drug properties that might otherwise remain hidden, providing a more targeted understanding of their effects, and aiding in the mitigation of their properties for drug repurposing [55]. We employed a network-based molecular model of PD to identify dysregulated molecular processes and pathways in PD. By analyzing differentially expressed genes and constructing a molecular network, we gained insights into the underlying pathophysiology of PD and its associated complications. This molecular network served as a basis for proposing novel drug combinations that have the potential to positively influence disease progression in PD patients.

By assessing the distances between drug targets and proteins in this PD molecular network, we identified drug clusters that showed significant associations with PD-related biological processes. We compared prescribed and non-prescribed drugs targeting the same biological processes, based on their proximity scores. Selective serotonin reuptake inhibitors (SSRIs) and vascular endothelial growth factor (VEGF) inhibitors were strongly represented in our final network, aligning gene targets of the pathophysiological processes angiogenesis and inflammation, both of which are highly relevant for complications of PD treatment.

VEGF inhibitors have been investigated for their potential to address peritoneal fibrosis [56,57]. Among these, bevacizumab has been studied for its anti-fibrotic potential in experimental PD, both as monotherapy and in combination with everolimus [58,59]. The VEGF inhibitors ranibizumab and aflibercept have been widely used in macular degeneration [60], an age-related pathology encompassing angiogenesis and vascular destabilization in the eye, which may be pathomechanistically similar to PD-related fibrosis indicated by the activation of pathways including TGF-β or IL-6 [61]. Except for bevacizumab, which has already been investigated for PD fibrosis, our analysis also identified the VEGF inhibitors ranibizumab, aflibercept, brolucizumab, and abicipar pegol as potential investigational molecules to address peritoneal fibrosis.

SSRIs have been investigated in vitro and in animal experiments for their potential anti-inflammatory properties [41,42,43,44]. Drugs like fluoxetine, dapoxetine, citalopram, and fluvoxamine, all categorized as SSRIs, directly target the *SLC6A* gene. In our analysis, these drugs exhibited significant proximity to the PD network. While traditionally prescribed as antidepressants (with dapoxetine being repurposed for the treatment of premature ejaculation [62,63,64]), patients treated with PD also often suffer from depression [65,66,67]. Nevertheless, traditional antidepressants were shown to exhibit anti-inflammatory properties, and anti-inflammatory drugs were shown to exhibit antidepressant effects [68]. Our molecular network analysis revealed SSRIs as potential anti-inflammatory agents, capable of modulating molecules involved in peritoneal inflammation, such as TLRs, NFκB, and IL-6 [69,70].

SSRIs have been investigated as potential targets for inflammation in various other contexts as well, including inflammatory bowel disease and inflammatory lung disease [71,72]. Notably, fluoxetine and citalopram specifically have demonstrated inhibitory effects on the TLR signaling pathway [73]. Furthermore, fluoxetine has been observed to inhibit NLRP3, a protein implicated in peritoneal fibrosis-related inflammation [74]. Thus, drug co-prescription offers a prospect of synergistic interactions between medications, potentially yielding additive therapeutic effects that surpass individual drug efficacy. Detecting such interactions may uncover new indications for existing drug combinations, broadening their therapeutic scope. The detailed study of co-prescription data, combined with pharmacological insights, holds potential for investigations of innovative treatment strategies in clinical trials. Furthermore, repurposing drugs that are already approved for other indications in kidney disease or failure—and for which clinical experience regarding dosing and elimination kinetics in dialysis patients exists—is more straightforward and may be facilitated more rapidly.

Our systematic approach involved identifying drug combinations that potentially modulate the same biological process through at least two distinct dysregulated molecules. We specifically focused on angiogenesis and inflammation, due to their significance in PD, closely associated with peritoneal fibrosis [75]. Moreover, we cross-referenced these combinations with drugs prescribed in our cohort of PD patients to prioritize clinically relevant options. Our research pinpointed several drugs. Those already prescribed in our cohort (e.g., pazopanib, celecoxib, meloxicam, rofecoxib, theophylline), as well as the currently not prescribed sunitinib, may exhibit potential to modulate proteins that are dysregulated in PD treatment. Mouse models of PD have demonstrated reduced angiogenesis and inflammation following celecoxib treatment [76]. Experimental evidence also suggests that theophylline can mitigate fibrosis in lung tissues by inhibiting the TGF-β signaling pathway, a pertinent topic in PD-related fibrosis [77]. We postulate a potential synergistic effect of combining celecoxib and theophylline, particularly given the TGF-β pathway’s involvement in PD-related fibrosis. While the evidence is limited, with only one experimental animal study and a single case report supporting it, there are indications that sunitinib may have positive effects on peritoneal membrane viability and could decrease VEGF expression in the dialysate [78,79].

Our analysis indicates that pairwise combinations of drugs, such as theophylline, fluoxetine, celecoxib, and amitriptyline, may target molecular pathways contributing to peritoneal fibrosis. Interestingly, fluoxetine and theophylline, as well as celecoxib and amitriptyline, have already been administered together in selected PD patients within our cohort. A detailed investigation of such subgroups may shed light on the potential synergistic effects of these drug combinations. However, preclinical experiments and clinical trials to carefully investigate the potential beneficial PD-related effects as well as the safety profile and adverse effects of these drugs, either alone or in combination, are needed before considering clinical application for new indications. Furthermore, the feasibility of these candidate therapeutic agents for clinical trials must be considered carefully in advance, especially as the elimination kinetics differs in kidney failure patients treated with PD. This is especially true for drugs with a narrow therapeutic window, such as theophylline, and for drugs that have been either withdrawn from many markets (e.g., rofecoxib) or that potentially increase the risk of cardiovascular events, especially on long-term use, such as -coxibes.

A limitation of this study is that the analysis relied on retrospective data from a single center. This database is well curated and continuously updated, but it still may be subject to inherent biases and constraints related to data collection and reporting. Additionally, while the proposed drug combinations offer promising insights, they require rigorous validation through preclinical and clinical studies to evaluate their safety, efficacy, and potential for adverse effects before clinical implementation. Therefore, this study is further limited by the lack of relation to clinical outcomes, external validation of our findings, and therefore, generalizability to other cohorts (especially with ethnic, demographic, or healthcare system differences), as well as the lack of prospective experimental or clinical testing of the identified candidate agents. The analyzed prescription points and data represent the documentation by medical personnel and are thus less prone to data entry errors; however, they do not correspond to treatment adherence.

## 5. Conclusions

In conclusion, this study highlights the potential of analyzing drug co-prescriptions as a strategy to address the complex comorbidities and complications associated with PD. By integrating long-term clinical data with molecular insights from a network-based model constructed using consolidated omics datasets, we identified commonly co-prescribed medications and proposed novel therapeutic combinations targeting dysregulated molecular processes in PD. Our findings also suggest that combinations including theophylline, fluoxetine, celecoxib, and amitriptyline may offer therapeutic benefit by modulating PD-related pathomechanisms. Additionally, specific drug classes (SSRIs, VEGF inhibitors) were found to pose anti-fibrotic potential. While further validation in experimental systems is required, our work provides a foundation for advancing personalized medicine in PD, offering promising directions for therapeutic innovation and drug repurposing to improve patient outcomes.

## Figures and Tables

**Table 1:** Demographics and PD therapy characteristics of the study cohort (*n* = 570).

	Mean ± SD/*n*(%)
Age (years) ^a^	54 ± 14
Sex (*n*, male/female)	330 (58%)/240 (42%)
Diabetes mellitus (*n* (%)) ^b^	132 (23%)
Etiology of ESKD (*n* (%))	
Chronic glomerulonephritis	108 (19%)
Diabetic nephropathy	72 (13%)
Polycystic kidney disease	64 (11%)
Vascular nephropathy	26 (5%)
Other or unknown	300 (53%)
Time on PD (months) ^a^	29 ± 22
PD modality (*n*, APD/CAPD/IPD) ^c^	224 (39%)/292 (51%)/54 (10%)
Endpoint at time of study end (*n* (%)) ^d^	
Kidney transplantation	219 (38%)
Transfer to HD	141 (25%)
Death	182 (32%)
Recovery of kidney function	8 (1%)
Transfer to another PD center	19 (3%)
Drug prescription timepoints	5835
Individual drugs ^e^	547
ATC code 4th level (drug category)	323
ATC code 5th level	764
Patients in decade 1/2/3 (*n* (%)) ^f^	137 (24%)/262 (46%)/171 (30%)

^a^ At PD start or first treatment at study center; ^b^ missing information of 4 patients; ^c^ initial PD modality; ^d^ lost to follow-up: 1 patient; ^e^ after curation (see methods); ^f^ 1: 1990–1999, 2: 2000–2009, 3: 2010–2022; ESKD: end-stage kidney disease, APD: automated PD, CAPD: continuous ambulatory PD, IPD: in-center intermittent PD, HD: hemodialysis, ATC: Anatomical Therapeutic Chemical.

**Table 2:** Drugs prescribed in PD patients with close proximity to the “PD disease network”.

Drug Class (ATC 2nd Level)	Drug	Drug Target (s)	Proximity Score *	*p*-Value	z-Score
Analgesics	Dihydrocodeine	OPRM1	0	0.0305	−1.916
	Hydromorphone	OPRM1	0	0.0305	−1.916
	Morphine	OPRM1	0	0.0305	−1.916
	Tramadol	OPRM1	0	0.0305	−1.916
Antibacterials for systemic use	Doxycycline ^#^	MMP7, MMP8, MMP13, MMP1, rpsB, […]	0.500	0.0174	−0.673
Antidiarrheals	Loperamide	OPRM1	0	0.0305	−1.916
Antiemetics	Ondansetron	HTR3A	0.600	0.0249	−0.425
Antihistamines for systematic use	Cetirizine ^#^	HRH1	0	0.0041	−1.916
	Desloratadine	HRH1	0	0.0041	−1.916
	Levocetirizine	HRH1	0	0.0041	−1.916
	Loratadine	HRH1	0	0.0041	−1.916
	Diphenhydramine ^#^	HRH1	0	0.0219	−1.916
Antihypertensives	Clonidine	ADRA2A, ADRA2C, ADRA2B	0.556	0.0077	−0.535
Anti-inflammatory and antirheumatic products	Celecoxib ^#^	PTGS2	0	0.0383	−1.916
	Meloxicam	PTGS2	0	0.0383	−1.916
	Rofecoxib	PTGS2	0	0.0383	−1.916
Antineoplastic agents	Pazopanib	CSF1R, KIT, LCK, FGFR3, FGFR1, PDGFRB, PDGFRA, FLT1, FLT4, KDR, ITK	0.273	0.0002	−1.238
	Capecitabine	TYMS	0	0.0050	−1.916
Cardiac therapy	Flecainide	SCN5A	0	0.0343	−1.916
Drugs for acid-related disorders	Sucralfate	PGA5	0	0.0021	−1.916
Drugs for constipation	Naloxegol	OPRM1	0	0.0305	−1.916
Drugs for obstructive airway diseases	Theophylline	PDE4A, PDE4D, PDE4B, PDE4C, PDE3A, PDE3B, ADORA1, ADORA2B, ADORA3, ADORA2A	0.600	0.0148	−0.425
Drugs for treatment of bone diseases	Denosumab	TNFSF11	0	0.0113	−1.916
Immunostimulants	Filgrastim	CSF3R	0	0.0298	−1.916
Immunosuppressants	Mycophenolic acid	IMPDH1, IMPDH2	0.600	0.0303	−0.425
Other nervous system drugs	Methadone	OPRM1	0	0.0305	−1.916
Psychoanaleptics	Citalopram	SLC6A4	0	0.0068	−1.916
	Escitalopram	SLC6A4	0	0.0068	−1.916
	Fluoxetine	SLC6A4	0	0.0068	−1.916
	Sertraline	SLC6A4	0	0.0068	−1.916
	Hydroxyzine	HRH1	0	0.0219	−1.916
	Paroxetine	SLC6A4	0	0.0343	−1.916
	Amitriptyline	SLC6A4, SLC6A2	0.500	0.0466	−0.673
	Duloxetine	SLC6A4, SLC6A2	0.500	0.0466	−0.673
	Milnacipran	SLC6A4, SLC6A2	0.500	0.0466	−0.673
	Venlafaxine	SLC6A4, SLC6A2	0.500	0.0466	−0.673
Psycholeptics	Melatonin	MTNR1A, MTNR1B	0	0.0080	−1.916
Urologicals	Finasteride	SRD5A2	0	0.0124	−1.916
	Sildenafil	PDE5A	0	0.0168	−1.916
	Tadalafil	PDE5A	0	0.0168	−1.916
	Vardenafil	PDE5A	0	0.0168	−1.916

* Proximity score: 0 = closest possible proximity (zero distance); ^#^ 2nd ATC code with different category available.

## Data Availability

All generated data are included in the manuscript and Supplementary Materials.

## Supplementary Materials

The following supporting information can be downloaded at https://www.mdpi.com/article/10.3390/jcm14113733/s1. Supplementary Figure S1: Workflow diagram.
