# Supplementary figures and images for: Integrative Analysis of Drug Co-Prescriptions in Peritoneal Dialysis Reveals Molecular Targets and Novel Strategies for Intervention
Source: J Clin Med. 2025 May 26;14(11):3733. doi: 10.3390/jcm14113733 (PMC12155629; doi:10.3390/jcm14113733)

Supplemental Figure S1: Workflow diagram

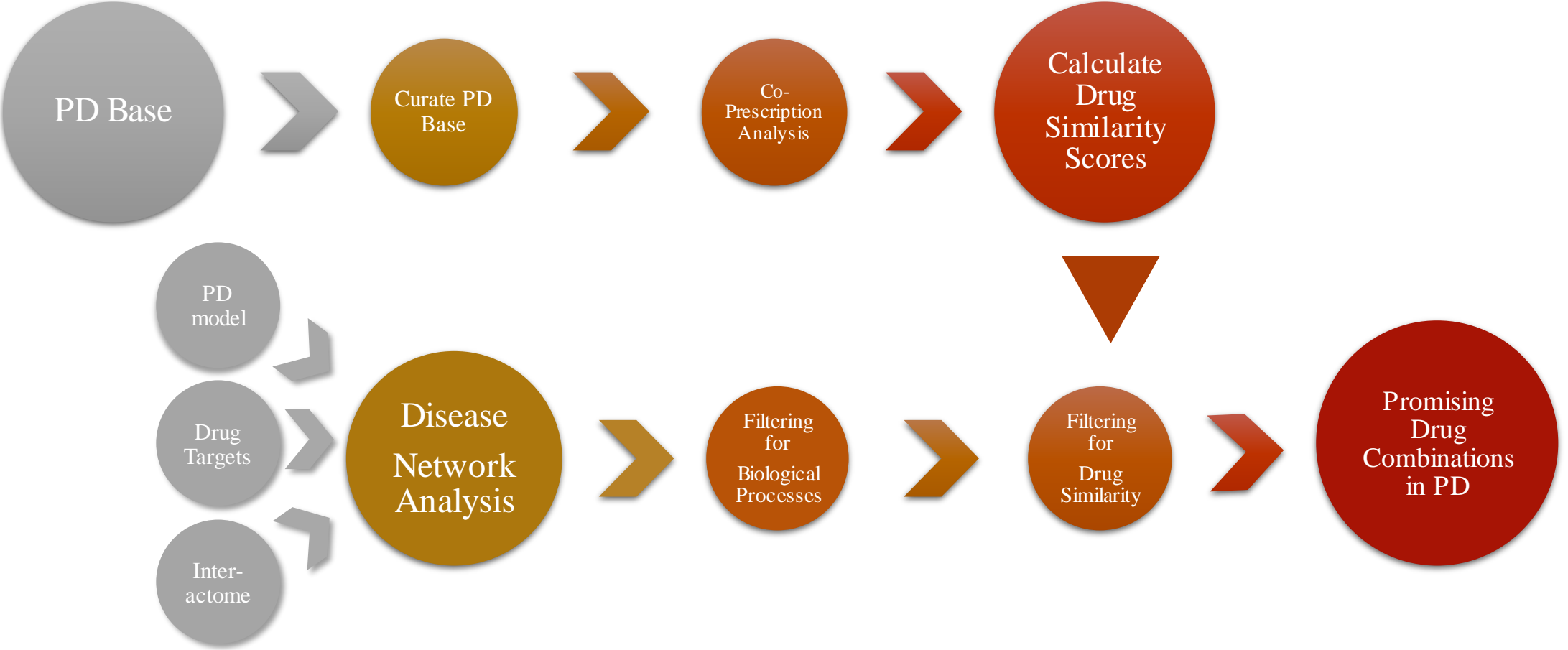

Supplement: Supplementary file 1 [file jcm-14-03733-s001.zip › jcm-3620316-supplementary/SupplFigS1.pdf]
